# Pro-Apoptotic Effect of Grape Seed Extract on MCF-7 Involves Transient Increase of Gap Junction Intercellular Communication and Cx43 Up-Regulation: A Mechanism of Chemoprevention

**DOI:** 10.3390/ijms20133244

**Published:** 2019-07-02

**Authors:** Antonella Leone, Cristiano Longo, Carmela Gerardi, James E. Trosko

**Affiliations:** 1 National Research Council (CNR), Institute of Science of Food Production (ISPA), Unit of Lecce, Via Prov.le Lecce-Monteroni, 73100 Lecce, Italy; 2 Food Department of Paediatrics and Human Development, Michigan State University, East Lansing, MI 48824, USA

**Keywords:** nutraceuticals, gap junction intercellular communication, Cx43 expression, grape seed Extract (GSE), MCF-7, apoptosis, chemotherapy, chemoprevention, antioxidants, connexins

## Abstract

Growing evidence suggests dietary antioxidants reduce the risk of several cancers. Grape seeds extracts (GSE) are a rich source of polyphenols known to have antioxidant, chemopreventive and anticancer properties. Herein, we investigated the in vitro effects and putative action mechanisms of a grape seed extract (GSE) on human breast cancer cells (MCF-7). The effects of GSE were evaluated on cell proliferation, apoptosis and gap-junction-mediated cell-cell communications (GJIC), as basal mechanism involved in the promotion stage of carcinogenesis. GSE (0.05–100 μg/mL) caused a significant dose- and time-dependent inhibition of MCF-7 viability and induced apoptotic cell death, as detected by Annexin-V/Propidium Iodide. Concurrently, GSE induced transient but significant enhancement of GJIC in non-communicating MCF-7 cells, as demonstrated by the scrape-loading/dye-transfer (SL/DT) assay and an early and dose-dependent re-localization of the connexin-43 (Cx43) proteins on plasma membranes, as assayed by immunocytochemistry. Finally, real-time-PCR has evidenced a significant increase in *cx43* mRNA expression. The results support the hypothesis that the proliferation inhibition and pro-apoptotic effect of GSE against this breast cancer cell model are mediated by the GJIC improvement via re-localization of Cx43 proteins and up-regulation of *cx43* gene, and provide further insight into the action mechanisms underlying the health-promoting action of dietary components.

## 1. Introduction

Breast cancer is the most commonly occurring cancer in women and the second most common cancer overall. There were over 2 million new cases in 2018 and the top 25 countries with the highest rates of breast cancer are in the West [1]. Environmental factors seem the major cause of breast cancer, among them are diet and lifestyle [2]. Although a more complex, multifactorial etiology is involved, a strong correlation between cancer and increased oxidative stress has long been established.

Nutraceuticals, non-nutritive dietary components, mainly phytochemicals with antioxidant activity such as polyphenols [3], might protect against human diseases related to oxidative stress, such as hypertensive and cardiovascular diseases, and cancer. Grape components, including by-products generated during wine processing as grape seeds, are rich sources of polyphenols [3,4,5,6,7,8,9].

It has been widely demonstrated that polyphenols, mainly flavonoids, are abundant in grape seeds and skin, and that they exhibit marked free-radical scavenger capacities and other important biological activities in vitro and in vivo [10]. The use of grape seed polyphenol extracts (GSE) and grape seed oil in foods, food supplements, additives, medicaments and cosmetic products and related patents are extensively known [11,12]. Many GSE phenolic compounds have shown suitable, albeit debated, bioavailability [13,14,15,16]; for this reason, studies on the action mechanisms underlying GSE effects on specific cell types are urgently required.

The most interesting constituents of grape seeds are monomeric phenolic compounds [(+)-catechin, (−)-epicatechin, and (−)-epicatechin-3-O-gallate], dimeric, trimeric, and tetrameric procyanidins, which are highly concentrated in the seeds of red grapes. Although the relative phenolic content can vary depending on the cultivar, vintage, grape ripening status, and processing [17,18], the classes of phenolic compounds are steady, flavonoids being the most representative. Both the purified compounds and the more complex GSE possess high antioxidant activity [19] and their cancer-preventive effects seem mainly due to that antioxidant ability, as has been demonstrated by different experimental approaches. However, a comprehensive understanding of the biological activities of grape seed components, including their mechanisms of action, has not as yet been achieved.

Previous in vitro and in vivo studies have shown that polyphenols modulate the activity of cell-signalling proteins, transcription factors and consequently mRNA expression including epigenetic microRNA (miRNA) regulation [20,21,22,23]. The cancer chemopreventive effects of GSE [20] and the proanthocyanidins it contains [22,23,24,25] were also related to its epigenetic-modifying properties, offering new perspectives on epigenetic regulation in cancer prevention. Still controversial [26] and although their precise underlying mechanism(s) are currently not well understood, epigenetic changes could play a major role on the health effects of dietary polyphenols. Altogether, these findings support GSE potential in cancer prevention and suggest that inflammation, cell proliferation, and apoptosis are affected by GSE, not only through antioxidant activity alone but also by gene expression modulation.

A cancer chemopreventive agent could be effective at any of the classically defined stages of carcinogenesis: Initiation, promotion, and progression, although such agents should intervene during the promotion phase, when initial premalignant/precursor lesions sometimes take a decade or so to proliferate and acquire invasive ability [25,26]. The promotion phase of carcinogenesis involves not only the mitogenesis of the “initiated” cell, but also the inhibition of apoptosis of those initiated cells [27]. The promotion phase, which can take decades to occur would seem to be the most efficacious strategy for dietary intervention to prevent cancers. In addition, once an initiated cell has become neoplastically transformed to invade and metastasize to another organ, it is no longer identical to the promoted “initiated” cell. Therefore, chemotherapeutic approaches must rely on additional strategies to stop the metastatic properties. Induction of either differentiation and apoptosis of the “cancer stem cell” in the tumour would seem to be the logical goal of chemoprevention and chemotherapy. Anticancer chemotherapeutic agents are somewhat effective at inhibiting cancer cell growth in vitro and in vivo, by triggering apoptotic events. There is abundant evidence showing that GSE can induce apoptosis in cancer cells, although the exact action mechanism is still not clear [23]. These kinds of studies must now be put into the context of knowing all tumours are a mixture of “cancer stem cells” and “cancer non-stem cells”. It is the “cancer stem cells” that must be targeted in order to prevent them from sustaining the growth of the tumour [28].

Since gap junctions have been shown to be involved in the apoptotic process [29] the prevention of the down regulation of gap-junctional intercellular communication (GJIC) by tumour promoting agents or the forced up-regulation of GJIC in non-communicating cancer cells would be the logical strategy to induce apoptosis. Several phytochemicals are able to induce apoptosis in cancer cells by enhancing gap-junctional intercellular communication (GJIC) [30,31,32,33,34]. Cancer cells usually exhibit dysfunctional GJIC, due generally to the lack of expression of connexin genes or the aberrant localization of connexin proteins [28,35], the structural components of the hexameric hemichannels that, when juxtaposed on adjacent cells, build up intercellular gap junctions [36]. As the tumour promoter agents exert their action by affecting cell-cell communication [37,38], several natural compounds, such as phytochemicals able to restore or enhance GJIC, although transiently, are able to allow cell-cell coupling and induce apoptosis through the passage of chemical signals [30,39,40,41,42,43]. Indeed, the induction of apoptosis by dietary factors in precancerous and cancer cells could offer an important chemopreventive mechanism, providing a physiological mechanism for the elimination of abnormal cell proliferation.

A growing amount of evidence has shown that several phytochemicals, including polyphenols, are able to exert their protective action, in cancer and non-cancer cells, through GJIC enhancement and changes in connexin localization and expression [30,32,44,45,46,47,48,49,50]. However, to the best of our knowledge a relationship between the protective effect of GSE and the GJ functions has not yet been established.

Connexins (Cxs), the structural proteins of the channel-forming gap junctions (GJs), are documented as tumour suppressor proteins as their re-expression into tumour cells decreases their tumorigenicity and reverses the transformed phenotypes of these tumour cells. Independent studies have shown tumour-suppressing effects of several Cxs [51,52]. Among these, Cx43 has been studied extensively because of its widespread expression [53]. The tumour suppressive activity of Cx43 is related to both, the cell-cell exchange of specific molecules through GJs and also via GJ independent pathways [54,55,56,57]. Altered expression, localization, and function of Cxs have been reported in human breast cancer tissues and in cell lines [58,59,60,61,62,63], suggesting that Cxs have tumor suppressive roles. Due to its positive correlation with improved disease outcome in breast cancer patients, Cx43 was also proposed as an independent predictor factor [64,65].

While the underlying mechanisms for the enhanced GJIC by these chemopreventive agents is not known in detail, oxidative stress induction of signal transduction has been hypothesized, which can alter gene expression, epigenetically, at the transcriptional, translational or posttranslational levels [66]. Focusing on breast cancer and GSE, some studies have demonstrated that GSE triggers growth arrest and cell death in human breast carcinoma cells [67] and inhibits cell migration in the highly metastatic human breast carcinoma cell line, MDA-MB231 [68].

The hypothesis that restoring the GJ function among tumor cells has the potential to suppress progression, migration, invasion, and metastasis of breast cancer cells and maybe other tumorigenic cells, has inspired the interest in GJs as novel therapeutic targets [69,70]. On the basis of this hypothesis, we intend to prove that the chemopreventive action of some compounds that passes through the pro-apoptotic activity, is mediated by the modulation of the gap junction intercellular communication and/or the related connexin proteins. The latter can be considered both a target for a first screening of the effectiveness of natural bioactive compounds or a possible target for the chemopreventive action in the lead compound screening in drug discovery. Although the chemopreventive effect of non-drugs dietary components through GJ modulation, could have a more meaningful impact on public health.

This work aims to clarify some mechanisms of action putatively underlying the promising health properties of GSE. Our hypothesis was that one of the action mechanisms underlying the antiproliferative and pro-apoptotic action of GSE on non-metastatic breast cancer cells is related to their ability to modulate GJIC and connexin expression. The vast literature about the multi-functional, multi-targeting action of GSE and the contained molecules, indeed, does not mention yet the gap junctions as target and mechanism of action involved in. Proving the involvement of GJ-proteins and/or GJ mediated intercellular communications in the action of GSE will allow us to better understand its anti-cancer action and maybe that of other natural compounds. In addition, this would allow us to do a further validation of the use the GJ-modulation ability as a target for basic screening for the anti-cancer action of several bioactive molecules.

## 2. Results

### 2.1. GSE Extraction, Characterization and Antioxidant Activity

Total phenolic content and antioxidant activity of grape seed (GS) polyphenolic extracts (GSE) obtained with two solvent mixtures are reported in Table 1.

The yield of phenolic compounds was significantly higher in the 80% methanol than in the 80% ethanol extract (1.64 ± 0.04 and 0.55 ± 0.02 mg of gallic acid equivalent, GAE/g of GS powder, respectively) confirming that methanol is an effective solvent for extracting phenolic compounds [71]. The antioxidant activity (AA) assayed in the hydroalcoholic extracts was higher in the GS extracted by methanol than ethanol (11.6 ± 0.5 and 3.3 ± 0.1 mol TE/100g GS), however, AA showed no significant differences between the extraction solvents, when GSE were expressed in terms of phenolic content (7.07 ± 0.54 and 5.87 ± 0.70 mol TE/g of GAE in 80% methanol and 80% ethanol, respectively). Given its higher extraction yield and easy solvent removal, the 80% methanol extract was used for subsequent experiments. HPLC analysis indicated that GS extracted using 80% methanol contained mainly gallic acid (6.2 ± 1.0 μg/mg of dried GSE), catechin (5.1 ± 1.3 μg/mg of dried GSE), epicatechin-gallate and epicatechin (3.2 ± 0.33 and 0.1 ± 0.02 μg/mg of dried GSE, respectively). Dried GSE was subsequently solubilized in 50% acetonitrile, a solvent less toxic to living cells, for subsequent experiments with MCF-7. The GSE antioxidant activity remained stable when polyphenolic extracts were solubilized in 50% acetonitrile; the AA indeed was always equal to or greater than the AA measured in GSE solubilized in methanol (data not shown).

### 2.2. Effect on Cell Viability

**Figure 1:**
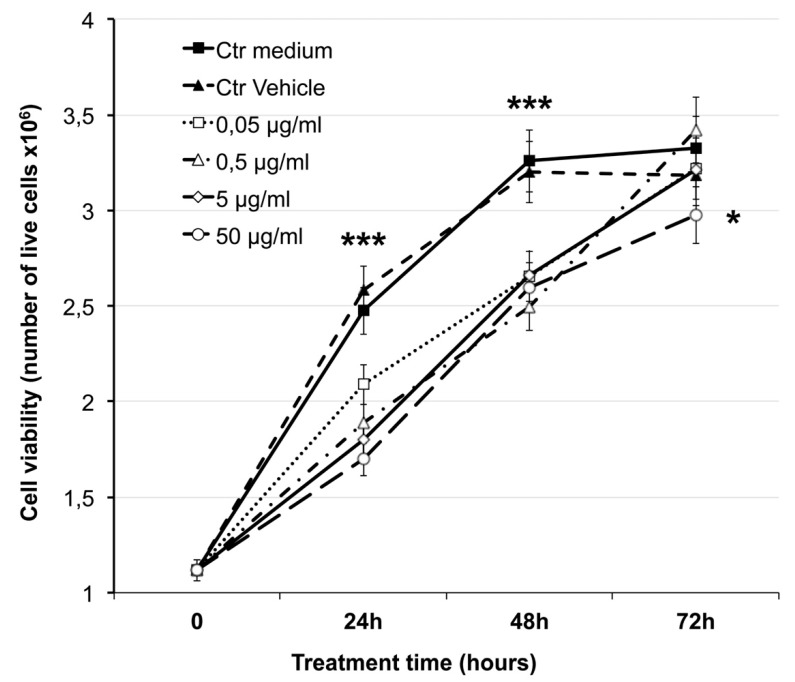
Time course and dose-dependent effect of GSE on MCF-7 cell viability. Concentration is expressed as μg of gallic acid equivalent (GAE)/mL of medium. Data are mean ± SD of three independent experiments in quadruplicate (* *p* ≤ 0.05, *** *p* ≤ 0.01).

In order to evaluate the effect of GSE on MCF-7 cells, dose-response and time-response experiments were set up. Figure 1 shows the effect of a large range of GSE concentrations (0.05–50 μg of total phenols (GAE)/mL), on MCF-7 cell viability assayed after 24, 48 and 72 h. A significant (*p* < 0.01) decrease in cell viability was evident after 24 and 48 h at all tested GSE concentrations as compared to the controls. The decline in cell viability was higher and dose-dependent after 24 h, while after 48 h the inhibitory effect of all tested concentrations was less consistent. Finally, after 72 h, cells completely recovered the ability to proliferate and no significant differences in cell viability were found, except at the highest tested concentration (50 μg/mL, *p* < 0.05). These results indicated higher bioactivity of GSE at lower concentrations and in the short term (24 h). Morphological changes were evident in MCF-7 cells treated with the higher GSE concentrations and for longer periods, including cell enlargement and epithelial-like appearance of the cell cultures (data not shown).

#### Apoptosis

**Figure 2:**
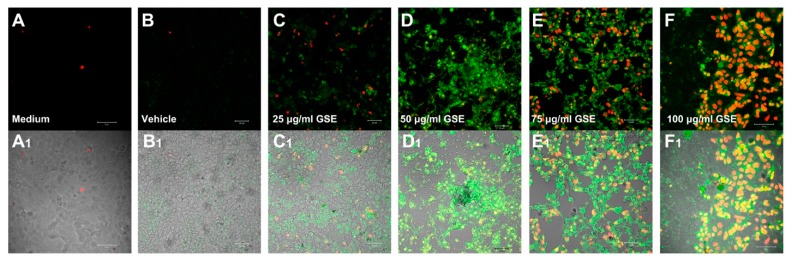
Confocal images of apoptosis detected by labelling with Alexa Fluor^®^ 488-conjugated Annexin V (green) and propidium iodide (red), in MCF-7 cells treated with 25, 50, 75 and 100 μg of GAE/mL GSE for 24 h and compared with untreated cells (medium) and cells treated with 0.025% acetonitrile as vehicle control (vehicle). (**A**–**F**) are the matching images from red and green channel fluorescence detectors; **A_1_**–**F_1_**, combination with the transmitted light images. Images shown are representative of three independent experiments, each done in quadruplicate. Bar is 50 μm.

**Figure 3:**
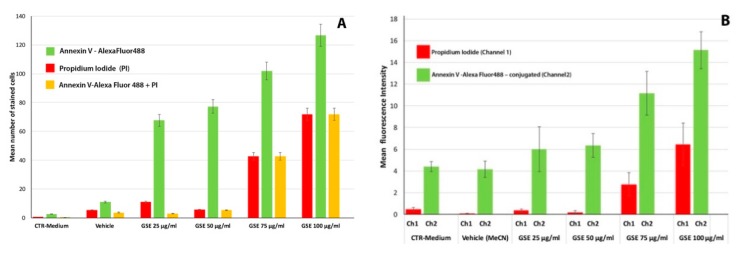
Quantitative analysis of apoptotic MCF-7 cells treated with different doses of GSE and stained with propidium iodide (PI) and Annexin V –Alexa Fluor488 – conjugated. (**A**) Mean of stained cells with PI (in red), Annexin V -AlexaFluor488 – conjugated (in green) and with both, PI and Annexin V-Alexa Fluor488–conjugated (in yellow) per field; each image captured a field corresponding to 106062.88 µm × µm of area (1048576 pixels) and about 209 ± 28 cells. (**B**) Mean intensity of fluorescence detected by the channel 1 (Ch1), corresponding to the PI emission fluorescence, and by channel 2 (Ch2) corresponding to AlexaFluor488 emission fluorescence; the analyses were made on the merged images (*n* = 11), 325.35 µm × 325.35 µm of area (x:1024, y:1024) with Zen software (Zeiss). Data are mean ± SD.

In order to verify whether the effect of GSE on MCF-7 viability was related to apoptosis, we evaluated the presence of apoptotic cells in cultures treated with the effective concentrations of GSE (25–50 μg GAE/mL). In addition, we also ascertained the nature of the cytotoxic effect at higher concentrations (75–100 μg GAE/mL). Figure 2 and Figure 3A show a significant dose-dependent increase in the number of MCF-7 cells undergoing apoptosis following treatments with all the tested concentrations of GSE, as evidenced by double labelling with propidium iodide (PI) and Annexin V immunodetection and confocal microscopy. Viable cells were negative for both PI and Annexin V-Alexa Fluor^®^ 488 staining, early apoptotic cells showed cytoplasmic green labelling (Annexin V-Alexa Fluor^®^ 488 staining) and were negative for PI, and late apoptotic dead cells displayed both Annexin V-Alexa Fluor^®^ 488 and PI labelling (co-localization). No, or very few apoptotic cells, were detected in the control cell cultures (Figure 2A–A_1_,B–B_1_). Figure 3A shows a very few number of apoptotic and necrotic cells in the cells treated with the vehicle only, that is compatible with a faint effect of acetonitrile. For this reason, and in order to take count any vehicle effect, the samples treated with vehicle only were considered as a control. GSE at 25 μg/mL (Figure 2C–C_1_) was able to trigger apoptosis, while the maximum detection of the green fluorescent Annexin V, indicating early apoptosis events, was evident in MCF-7 cells treated with 50 μg/mL (Figure 2D–D_1_). At higher concentrations (75 and 100 μg/mL, Figure 2E–E_1_,F–F_1_), cells in late apoptosis (green cytoplasm and red nucleus) were mainly detected.

The visual observations were confirmed by the quantitative analysis in Figure 3A,B showing an increase of the number of Annexin V positive cells with the increasing concentration of GSE and an increase of late apoptotic cells (co-localization of PI and AnnexinV), as compared to the total of apoptotic cells. Morphological changes are visible in MCF-7 cells treated with 100 μg/mL GSE, where apoptosis affects specific areas of the cell culture. At the higher concentration tested all the dead cells were also positive to Annexin V showing that the reduction of cell viability was due only to apoptosis and no necrotic effect of GSE was detected.

### 2.3. Effect on GJIC

The inhibition of cancer cell growth through apoptosis induction is a feature of many anticancer agents, although the exact mechanism is often still unknown. Several phytochemicals are able to induce apoptosis in cancer cells by GJIC enhancement [30,32,72] or cx43 over-expression [73]. In order to explore the hypothesis that GJIC is involved in chemiotherapeutic or chemopreventive action of GSE and apoptosis trigger, we tested GJIC functionality, Cx43 protein localization, and *cx43* mRNA expression in MCF-7 cells treated with different concentrations of GSE.

#### 2.3.1. GJIC Functionality

**Figure 4:**
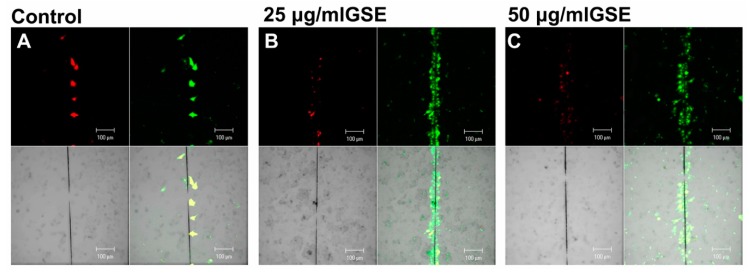
Gap-junction-mediated cell-cell communications (GJIC) functionality assessed by scrape-loading/dye-transfer (SL/DT) assay in MCF-7 cells treated with GSE (25 or 50 μg GAE/mL) for 30 min. Each image is representative of three independent experiments, each done in triplicate (*n* = 27) and shows the single channel detection of the cells containing the non-gap junctions (GJs) -permeant Rhodamine dextran, 10,000 MW (red), or the GJ-permeable Lucifer Yellow CH (green) or transmitted light images (in grey); at the bottom right of each image is the match of all channels. (**A**) Non-treated cells (controls); (**B**) cells treated with 25 μg/mL GSE; (**C**) cells treated with 50 μg/mL GSE. Bars 100 μm.

**Figure 5:**
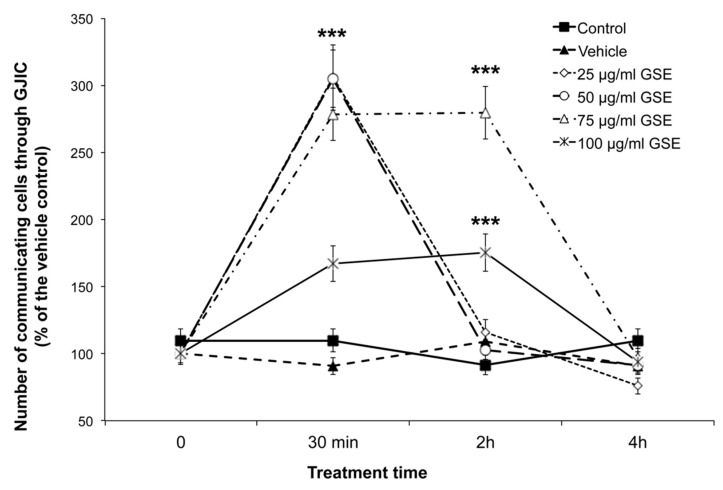
Dose- and time-response of GSE treatments on gap junction intercellular communication (GJIC) in MCF-7. Data are expressed as percentage of the relative controls and are expressed as the mean ± SD of three independent experiments (*n* = 6). *** *p* ≤ 0.01.

Figure 4 shows representative images of the scrape-loading/dye-transfer (SL/DT) assay used to detect the presence of functional gap junctions. An excellent correlation between GSE treatments and the increase in cell-cell communication mediated by gap junctions was observed at 30 min of GSE treatment. The enhancement of GJIC was a transient phenomenon. Indeed, no changes in GJIC functionality were detectable at 24 h of GSE treatment, at all the tested GSE concentrations, although the apoptotic effect was evident after 24 h. Like most tumour cells, MCF-7 were non-communicating cells [32], as shown in Figure 4A. In the presence of GSE, a rapid increase in communicating cells was detected as GJ-coupled green fluorescent cells (Figure 4B,C). Figure 5 shows the quantitative analysis of communicating cells treated with GSE at 25, 50, 75 and 100 μg GAE/mL, after 30 min, 2 h and 4 h of GSE treatment. A transient and significant increase in cell–cell communication mediated by gap junctions was observed over time at all GSE concentrations. At 25 and 50 μg GAE/mL, a transient GJIC increase, of three-fold as compared to the control, was measured after 30 min of treatment, but not after 2 and 4 h. The two higher concentrations of GSE (75 and 100 μg GAE/mL) were able to exert a prolonged effect on GJIC, which was detectable after 2 h of treatment, although the highest GSE concentration (100 μg GAE/mL) increased GJIC to a lesser extent, probably because of its toxicity. The GJIC increase was a transient phenomenon since no significant difference, as compared with the controls, was detectable after 4 h of treatment at all the tested GSE concentrations (Figure 5). The rapid increases in the GJIC functionality indicates that the main mechanism should involve a change in the functionality of connexin proteins already present in the cells and/or the opening of abnormally closed gap junction channels.

#### 2.3.2. Western blot Analysis of Cx43 Protein

**Figure 6:**
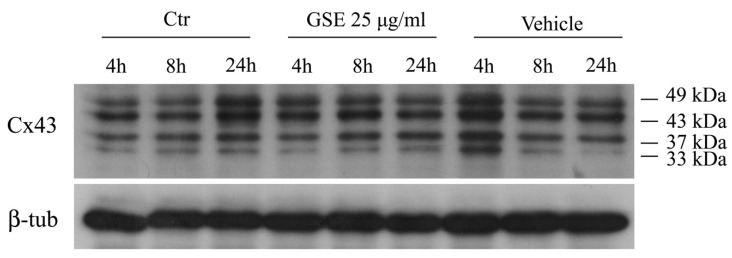
Western blot analysis of Cx43 protein. MCF-7 cells were incubated with 25 μg/mL GSE for 4, 8 and 24 h, and 15 μg of extracted proteins were separated by SDS–polyacrylamide gels and transferred onto nitrocellulose membranes. The membranes were probed with the anti-Cx43 antibodies and ECL detection. Beta-actin was used as a control.

A preliminary assessment of Cx43 protein in MCF-7 cells treated with 25 μg/mL of GSE was done at 4, 8 and 24 h of treatment, untreated cells and cells treated with vehicle only were included as controls at each time. Figure 6 shows that in all the tested samples, four proteins bands positive to anti-Cx43 antibody were detected. Other than the Cx43 band corresponding to its regular MW, namely 43kDa, a band at 49 kDa, corresponding to the hyper-phosphorylated Cx43 proteins and two further bands at 37 and 33 kDa, were detected. The immunoreactive 37 and 37 kDa protein bands could represent cleavage products of not functional proteins or intermediate polypeptides during Cx43 synthesis. The endocytic trafficking of Cx43 during the polypeptide biosynthesis and localization as well as the degradation processes of not functional Cx43, have been widely documented [74,75,76,77,78]. In our case, however, western blot analysis give us no supplemental information since there were no indicative differences in both, the number of bands and the detected amount of Cx43 proteins. Indeed, the western blot analysis give a static picture of the status of proteins. We deduced that the possible rapid (less than 4 h) changes in the Cx43 phosphorylation status and possibly in the Cx43 localization could correspond to a very little change in the protein content and band distribution that are hard to detect by western blot analysis. As a consequence, protein cell localization and expression of RNA of cx43 would have been more relevant information and better detected by means of immunolocalization and real-time PCR, rather than by western blot.

#### 2.3.3. Cx43 Localization

**Figure 7:**
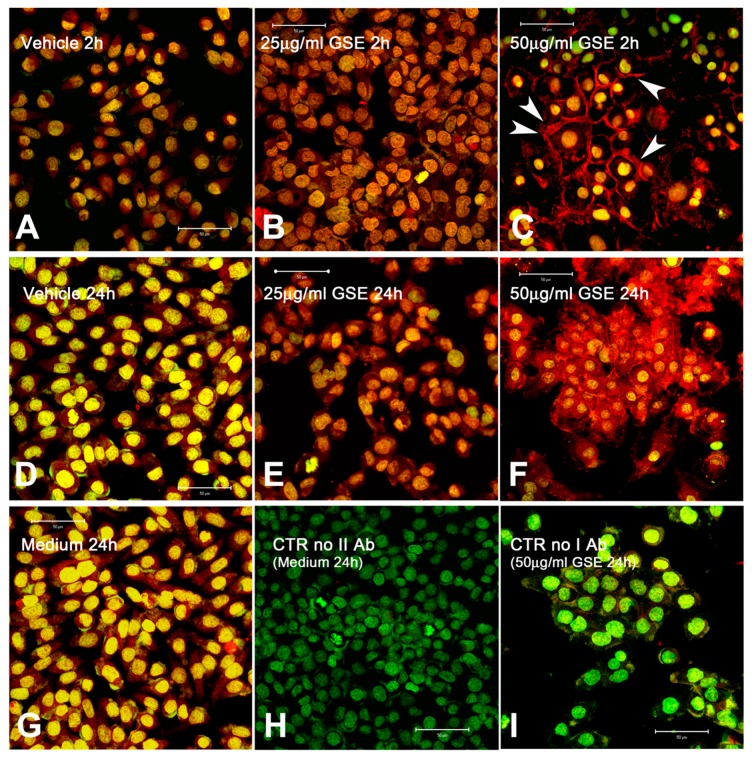
Localization of Cx43 proteins in MCF-7 cells by indirect immunofluorescence using anti-Cx43 antibody conjugated to Alexa Fluor^®^ 633 (red) and counterstained with SYBR green nuclear dye (green). MCF-7 cells were treated with 0.025% v:v acetonitrile for 2 h (**A**), and for 24 h (**D**), with 25 μg/mL GSE for 2 h (**B**) and for 24 h (**E**), and with 50 μg/mL GSE for 2 h (**C**) and for 24 h (**F**). Untreated MCF-7 cells were always included, here (**G**) a representative image related to 24 h. Technical controls without the secondary antibody (**H**) or without the primary antibody (**I**) were also included. In the controls (medium and vehicle), Cx43 is recognized in the cytoplasm (red). Treatment with 25 μg/mL GSE induces a weak increase in Cx43 immunolabelling at both 2 h and 24 h. A dramatic change in Cx43 localization and plaque formation is induced after 2 h of treatment with 50 μg/mL, and accumulation of immunolabelled Cx43 is visible after 24 h of treatment. Laser scanning confocal microscope images are results of merged channels (red and green) and were seen under 40 × Neo-Plan objective lens, pinhole = 0.90 μm. Control cells treated with vehicle (**A**,**D**), or medium only (**G**). Arrows indicate plaque formation. Images are representative of three independent experiments. Bars = 50 μm.

In order to verify the putative posttranslational modifications of connexins able to modulate their functionality, the level and cell localization of the most diffuse connexin protein, Cx43, were investigated. Figure 7 shows the immunolocalization of the Cx43 protein in MCF-7 cells treated with different GSE concentrations for 2 h or 24 h. In the controls (Figure 7A,D,G), Cx43 is recognizable as diffuse intracellular staining, including the nuclear zone, as indicated by the co-localization of red labelled Cx43 and SYBR green dye, visible as yellowish nuclei. This indicated a stable presence of low levels of intracellular Cx43 proteins, putatively unable to reach the cell membrane. Treatment with GSE (25 μg GAE/mL) induced a weak increase in Cx43 immunolabelling at both 2 h and 24 h (Figure 7B,E). Higher concentrations of GSE (50 μg GAE/mL) were able to induce a dramatic change in Cx43 localization after 2 h and an accumulation of immunolabelled Cx43 after 24 h of treatment (Figure 7C,F, respectively). Indeed, after 2 h, cells treated with 50 μg/mL of GSE displayed an intense Cx43-Alexa Fluor^®^ labelling (red fluorescence) on the cellular peripheral zone (arrows in Figure 7C). The labelling corresponded to cell-cell contact regions as confirmed by comparison of phase-contrast images (not shown). Thus, in the presence of GSE, the distribution of the immunolabelled Cx43 switched rapidly from endocellular localization to the plasma membranes. Together with the functional results regarding cell-cell communication (Figure 5), Cx43 localization suggested that, in the presence of GSE, functional gap junction plaques are properly formed and are able to quickly allow intercellular coupling. The subsequent fate of the Cx43 protein, involving the endocytic vacuole, determines the endocellular localization and accumulation of Cx43. After 24 h of GSE treatment, indeed, MCF-7 cells exhibited strong immunofluorescent Cx43 labelling in both endocellular and peripheral regions (Figure 7F). These data suggest that Cx43 proteins present in MCF-7 cells are not functional as GJ-channels, they showed an aberrant cytosolic localization and seem not able to correctly translocated and positioned on the cell membrane to form functional gap junction plaques. The endocytic trafficking and degradation processes of not functional Cx43, also through internalization of complete gap junctions, have been widely documented [74,75,76,77,78].

In addition, our results seem to indicate that GSE, at suitable concentrations, is able to induce a short-term effect by re-localizing the pre-existing pool of Cx43 proteins, as well as the possible neo-synthesis of Cx43. The longer-term effect (24 h) of GSE treatment at 50 μg/mL was an increased occurrence of Cx43, which was largely located on the cell membrane and accumulated at cytosolic level, possibly due to the intensified synthesis. Many phytochemicals are able to induce changes in connexin functionality by re-localization, generally mediated by specific phosphorylation events [30,47]. The difference in cell communication capacity is assumed to be due to the phosphorylation status of Cx43, which is in part responsible for its ability to form functional hemichannels, intercellular complete channels, and functional plaques (clusters of GJ-channels) and for its consequent intracellular localization. The majority of connexins are phosphorylated at specific sites, and the different phosphorylation sites of Cx43 have been widely studied [75,79].

#### 2.3.4. Cx43 mRNA Expression

**Figure 8:**
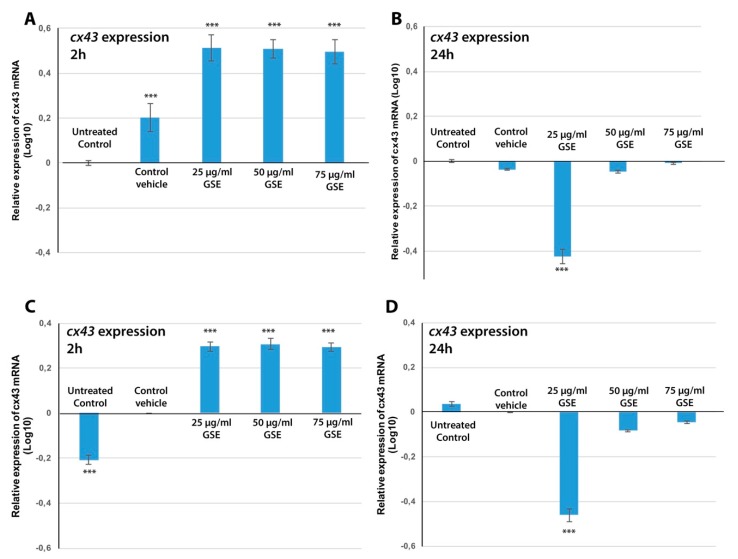
Relative quantification of the expression of *cx43* mRNA in MCF-7 cells treated with different amounts of GSE for 2 h (**A**,**C**) or for 24 h (**B**,**D**). Relative quantification was calculated using the ΔΔCt method and expressed as Log_10_ of relative expression of cx43 mRNA as referred to untreated control (**A**,**B**) and to control vehicle (**C**,**D**). The mean of each triplicate well is plotted and the error bars represent SE. *** *p* ≤ 0.01.

In order to test the hypothesis that GSE could affect the connexin expression the quantitative evaluation of *cx43* gene was performed. Our results demonstrated that GSE also affects Cx43 mRNA expression. Figure 8 shows Cx43 gene expression evaluated by real-time quantitative PCR performed on MCF-7 cells treated with doses of GSE effective on cell viability and GJIC functionality (25, 50 and 75 μg/mL) for 2 h or 24 h. After 2 h of treatment, all three GSE concentrations were able to significantly increase *cx43* expression in MCF-7 cells up to three fold, as compared to the vehicle control and up to five fold as compared to untreated cells. The relative level of *cx43* mRNA in cells with only medium (control) resulted down regulated when data were referred to the expression in vehicle treated cells (Figure 8C). No significant difference among the three tested GSE concentrations was detectable in their ability to switch on the induction of *cx43*, indicating an on-off effect. After 24 h (Figure 8B,D), only treatments with lower GSE concentrations (25 μg GAE/mL) were able to severely alter the levels of *cx43* mRNA, with a significant drastic *cx43* down-regulation mainly evident in the presence of 25 μg GAE/mL compared with 50 μg GAE/mL treatment. The higher GSE concentrations (75 μg GAE/mL) did not significantly affect *cx43* expression. This is consistent with our results on apoptosis detected after 24 h of treatment with higher GSE concentrations. The apoptotic process, indeed, slows down the synthesis of most proteins, including connexins.

## 3. Discussion

The objective of the present study was to evaluate the effect of GSE in an in vitro breast cancer model, namely MCF-7 cells, in order to determine if the involved mechanisms of action is related to the GJIC modulation and connexin43 expression.

A large majority of published in vitro studies focused on breast cancer and/or estrogen receptor biology have used the MCF-7 human breast cancer cell line. MCF-7 is indeed a commonly used breast cancer cell line, proved to be a suitable in vitro model for breast cancer investigations, including those regarding putative anticancer drugs [80]. Since MCF-7 is a poorly-aggressive and non-invasive cell line, normally being considered to have low metastatic potential [81], it results a good cell system to assay chemopreventive potential of chemicals. Currently, a large body of evidences identifies connexins as tumor suppressors in breast cancer pathogenesis [82]. In many studies [30] including ours [32,44], it was demonstrated the involvement of GJ functionality in the anti-proliferative action of several natural compounds, in MCF-7. It was also demonstrated that in MCF-7 cells GJ function could influence the cytotoxicity of chemical drugs such as adriamycin [83]. For all those reasons, MCF-7 appears as a suitable system for the evaluation of chemopreventive and therapeutic effect of natural compounds.

Grape seed extracts and particularly the protoantocyanidin they contain, are known to have strong antioxidant capacity and anticancer activity also affecting several key cancer–associated genes [3,8,24]. GSE have been also considered as chemopreventive agent against breast cancer due to their demonstrated anti breast cancer action thorough suppression of aromatase/estrogen biosynthesis [84,85], although the action mechanisms are not all completely known. In our study, a first circumstantial evidence was that the cytotoxic and/or active effect of GSE on MCF-7 was strictly dependent from the polyphenol concentration and the time of treatment. Indeed, GSE affected MCF-7 cell viability at higher extent at the shorter considered treatment time (24 h), when a consistent dose-response effect was evident (Figure 1), while high concentrations or longer time of actions lead to not consistent results. This could also explain the inconsistent results for single polyphenols or complex extracts on cell viability, when a single concentration or exposure time, or different cell types are considered [67,68,86]. In our experimental conditions a pro-apoptotic effect of GSE on MCF-7 was established at concentrations ranging from 25 to 75 μg GAE/mL (Figure 2 and Figure 3). These data confirmed the selective induction of apoptotic cell death reported for a grape seed proanthocyanidin extract, which showed cytotoxicity towards some human cancer cells, including MCF-7, while enhancing the growth and viability of the normal cells examined [87]. In addition, GS proanthocyanidins, including the dietary form, led to in vitro and in vivo apoptosis, and inhibited tumour growth and metastasis of highly metastatic breast cancer cells through disruption of the mitochondrial pathway and increased activation of caspase-3 [88]. In addition, GSE synergistically enhanced the cytotoxicity of doxorubicin on human breast carcinoma cells [67]. Finally, it was showed that GSE regulates the activity of transcription factor related to the activity of core promoter of surviving, decreasing the gene expression of survivin [89].

Established that GSE reduce cell viability in our breast cancer model, MCF-7, by inducing apoptosis, we tested the effect of GSE on the GJ functionality in order to verify a putative causal relationship between GJ functionality improvement and apoptosis. Our results demonstrated that GSE was able to modulate cell-cell communication via GJIC in a dose-dependent and time-dependent manner (Figure 4 and Figure 5). Indeed, a rapid and transient (within 30 min of GSE treatment) increase of GJ functionality was recorded that was not compatible with the de novo synthesis and the subsequent correct localization of GJ structural proteins in membrane GJ-channels. The rapid GJIC enhancement indicates that the main mechanism involved in the early increase in GJ functionality could be a change in the functionality of connexin proteins already present in the cells and/or the opening of abnormally closed gap junction channels. The antioxidant role of GSE component could be pivotal in this first cell response. The level of ROS (reactive oxygen species) production in in vitro carcinoma cells is at a high rate as well as many tumours in vivo appear to be under persistent oxidative stress. In addition, in cancer cells the redox balance is importantly modulated by internal and external factors such as hypoxia; breast tumour rapidly outgrows, leading to glucose deprivation and hypoxia as well as to oxidative stress within the MCF-7 cell line, although it does not cause oxidative stress in nontransformed cell lines [90,91]. The presence of high level of ROS lead also to cell proliferation and to the activation of mitogen-activated protein kinases (MAPKs) [92].

In our experiments, the Cx43 immunolocalization in MCF-7 cells treated with GSE, demonstrated a change of cell localization and a time-dependent accumulation and internalization of Cx43 immuno-stained proteins. MCF-7 cells treated with 50 μg GAE/mL of GSE after 2 h of treatment showed a localization on the external profile of cells while after 24 h an accumulation in the internal membranes was evident (Figure 7). The endocytic trafficking and degradation processes of Cx43, also through internalization of entire gap junctions, have been widely documented [74,75,76,77,78]. Overall, our data suggest that Cx43 proteins are present in MCF-7 cells, but they are not correctly translocated and positioned on the cell membrane to form functional gap junction plaques, showing an aberrant cytosolic localization. This could confirm the hypothesis that one of the first response to GSE treatment is the conformational change of Cx43 inactive as GJ channel, maybe through phosphorylation events also mediated by the antioxidant activity exerted by of GSE components [30,47]. However, it was also reported [93] that the induced Cx43 overexpression in MCF-7 exerted tumor-suppressing action not only via GJ dependent but also via GJ independent pathways. For example, the enforced expression of Cx43 increased MCF-7 sensitivity towards artesunate treatment, an anti-malarial drug, and here we cannot exclude a similar GJ independent effect. In our system, new experiments are needed in order to be able to establish if GJ independent mechanism are involved.

Due to the rapid turnover of connexins [74], the persistence of the increased GJ functionality suggests that an enhancement in connexin expression (mRNA and proteins) could also be involved. In order to verify a de novo synthesis of Cx43 possibly induced by GSE, the expression of Cx43 mRNA was quantitatively assayed. In general, the reduced expression of connexins and/or absence of GJ intercellular communication (GJIC) was associated with tumour phenotype. Connexin43 had been found to be downregulated or poorly expressed in both MCF-7 and MDA-MB-231, a highly metastatic human breast cancer cell line, at both protein and mRNA levels [94,95]. A reduced Cx43 expression, indeed, has been proposed to be used as an independent marker for the detection of breast tumours [94].

Our results demonstrated a time- and dose-dependent modulation of *cx43* gene expression during GSE treatment. GSE, at all tested concentrations, at 2 h of treatment, was able to three-fold increase the expression of Cx43 mRNA as compared to vehicle control. The up-regulation effect was transient, indeed, after 24 h of GSE treatment, a down-regulation of cx43, mostly evident in cells treated with lower GSE concentration, was detectable. It was clear that one of the first effects of GSE treatment was the transient enhancement of GJIC via re-localization of pre-existing Cx43 proteins together with the induction of expression of Cx43 mRNA. The induced GJ functionality and induced expression of Cx43 could lead to apoptosis MCF7 cells. Actually, it was shown that Cx expression enhances apoptosis induction in HeLa cells, in a Cx-type dependent manner [96], suggesting that this effect could be due to the transfer of pro-apoptotic signals through the gap junctions. It was also found that the over-expression of cx43 was able to enhance drug sensitivity in human glioblastoma cells [73], by decreasing expression of the bcl-2 protein and significantly enhancing apoptosis during exposure to chemotherapeutic drugs, and interestingly, the action of cx43 seemed not to be directly related to GJIC [73] as was also shown in MCF-7 cells [93]. A so-called bystander death, induced by the spreading of cell-killing signals through GJs from cells undergoing apoptosis to surrounding cells, has been described for several apoptosis-inducing conditions. In our study, cx43 expression correlated well with GJIC functionality and Cx43 protein levels and localization data, indicating that the first step of GSE-induced GJIC enhancement could pass through the functionalization of pre-existing aberrant Cx43 proteins and then in the early induction of the cx43 gene. The first event results in changes in the cell localization of Cx43 proteins and the formation of functional GJ-plaques (Figure 4, Figure 5 and Figure 7C, respectively). Induced cx43 mRNA then undergo protein synthesis, vesicular transport, oligomerization, plasma membrane localization and degradation, participating in endomembrane trafficking and accounting for their intracellular localization. This phenomenon is transient and within 24 h the cx43 mRNA level drops below the control levels, by which time apoptosis events are already occurring in MCF-7 cells treated with all the GSE concentrations and the synthesized Cx43 protein is internalized in endocytic vacuoles (Figure 2 and Figure 7F). Changes in connexin expression profiles during GSE treatments could be related to epigenetic events. For example, it was demonstrated that the reduction in Cx26 expression in hepatocarcinoma was a consequence of its promoter hypermethylation. Furthermore, other events have been related to down-regulation of connexin expression, such as inappropriate phosphorylation pattern and aberrant subcellular localization [92,97].

Overall, although with basic experiments, this study is able to explain that the chemopreventive action of GSE components involves GJ and Cx43 expression. Even though we are not able to clearly indicate if only a GJ dependent or also GJ independent action of Cx43 is involved, we laid the foundations for an additional action mechanism of GSE components based on GJ involvement, that corroborates GSE as chemopreventive agent in breast cancer.

These results could be also considered in view to develop a new food design concept aiming to improve both human and environment health [98]. Indeed, the food industry, such as the wine sector, generate a great quantity of by-products whose storage, transformation, or elimination poses problems both in ecological and economic terms. For this reason, the recovery of valuable compounds present in food industry by-products could be an interesting advancement in maintaining environmental equilibrium and the human health.

## 4. Materials and Methods

### 4.1. Source of Material

Grape pomace from a red grape variety (*Vitis vinifera* L., var. Negroamaro) was collected directly from the local winery L’Astore Masseria (Lecce, Italy). Fresh pomace from pressed grapes, previously submitted to a maceration process, was directly recovered after winemaking. Whole pomace was dried in the air in a thin layer at room temperature for several days. When dried, the seeds were easily separated from the skin and stems, sieved, washed two times in distilled water, and further dried in a vacuum oven (SalvisLab, Industrie-Ost CH-6343 Rotkreuz, Switzerland) at 40 °C maximum. The grape seed (GS) moisture content was 5.5%, as determined by drying the seeds at 40 °C to constant mass. Cleaned and dried GS were placed into plastic bags in the absence of oxygen, sealed and stored in the dark, at 4 °C, until use.

### 4.2. Extraction

Dried GS were crushed in a blender (Waring, Torrington, CT 06790, USA) for 2 min, using 15 s pulses, to avoid heating the sample. GS-extract (GSE) was prepared by suspension of GS powder in 80% ethanol/20% H_2_O or 80% methanol/20% H_2_O in a ratio of 1:8 (w/v); the mixture was stirred by rotation at 14 rpm for 30 min, at room temperature protected from light. The insoluble powder was eliminated by centrifugation (2800× g for 10 min), the supernatant was filtered on Miracloth (Calbiochem^®^, Darmstadt, Germany) and the extracts were concentrated by rotary evaporation under vacuum at ≤40 °C. Lastly, the concentrated GSE was lyophilized in a freeze drier (Freezone 4.5L Dry System, Labconco Co. Thermo Scientific, Kansas City, MO 64132-2696) and stored at 4 °C until use. The dry residue was re-solubilized in 50% (v:v) acetonitrile/H_2_O, clarified by centrifugation and the supernatant was analyzed, as described elsewhere. All of the aforementioned operations were performed in light-protected conditions and at controlled temperatures in order to avoid polyphenol degradation [99].

### 4.3. Total Phenols

Total phenolic compounds in the GSE were assayed colorimetrically by the Folin–Ciocalteu method, as modified by [100]. A standard curve with gallic acid was built and the content of phenolics was expressed as gallic acid equivalents (GAE) per g of GS powder. In this work, the quantities of GSE used refer to their total phenolic content expressed as GAE.

### 4.4. HPLC Analysis

Quantification of flavonols was done by HPLC using gallic acid, (+)-catechin and (−)-epicatechin (from Fluka and Aldrich Chemical, Buchs, Switzerland) as external standards. The chromatographic system consisted of a Shimadzu LC-6A model (Shimadzu, Tokyo, Japan), fitted with a μBondapack (Waters Corporation, Milford, MA 01757, USA) C18 column (300 × 3.9 mm I.D.). The injection system used was a 20-μL-sample loop. Detection was performed using a UV-visible Spectrophotometer SPD-6AV set at a wavelength of 280 nm. Elution was carried out at a flow rate of 1.0 mL/min. The binary mobile phase consists of (A) acetonitrile: 4.5% formic acid (1:9) and (B) 4.5% formic acid. The gradient was as follows: From 10% B to 44% in 1.60 min, from 44% to 100% in 56.60 min and kept for 5 min. The compounds were quantified using a Shimadzu C-R4A Chromatopak data processor at a chart speed of 2.5 mm/min. The content of compounds was estimated by the external curve of each of the flavanols, gallic acid, catechin and epicatechin.

### 4.5. Antioxidant Activity

The total antioxidant activity of GSE was assayed by the TEAC (Trolox Equivalent Antioxidant Capacity) method [101] based on the scavenging of the blue/green ABTS radical [2,2′-azinobis-(3-ethyl-benzotiazolie-6-sulfonic acid)], (Fluka Chemie GmbH, Buchs, Switzerland), that is converted into a colourless product. Appropriate blanks with suitable solvent were run in each assay and different concentrations of Trolox (Sigma, Buchs, Switzerland) were used to build a standard curve. The antioxidant activity was expressed as mol of Trolox equivalents (TE) per gram of GSE.

### 4.6. Cell Cultures

The breast cancer cell line, MCF-7, containing the estrogen receptor, was obtained from the European Collection of Cell Cultures (ECACC, London, UK). The cell line was routinely grown as described in [44].

### 4.7. Cell Viability and Treatments

Cell viability was assessed by the Trypan Blue (Invitrogen™, Carlsbad, CA, USA) dye exclusion assay. MCF-7 cells were seeded at 0.5 × 10^6^ cells/mL in 35 mm plates or 24 well plates in complete medium (RPMI-1640 medium supplemented with 10% FBS, 2 mM glutamine (GLN), 50 U/mL penicillin G, 50 μg/mL streptomycin, all from GIBCO (Thermo Fisher Scientific, Waltham, MA 02451, USA) and treated as described below. For the qualitative cell viability analysis, MCF-7 cells were seeded in 35 mm plates; after 24 h, the culture medium was replaced with complete medium containing GSE at the following concentrations: 0.05, 0.5, 5 and 50 μg of GAE/mL of medium. A single dose of GSE, at the different concentrations, was administered to the cells and the cell viability was assayed at 24, 48 and 72 h after treatments, by trypsinization. Viable cell counting was performed by the Countess^®^ Automated Cell Counter system (Invitrogen, Carlsbad, CA, USA). For the negative controls, the test compounds were replaced with the vehicle (0.025%, v/v acetonitrile) or medium only, and controls were included on each plate set. The cells were incubated at 37 °C and 5% CO_2_ and assayed for vitality after 16 h. Three independent experiments were performed in quadruplicate, for each treatment and controls with vehicle and medium only. The cell viability was also measured by MTS assay that confirmed the results obtained by Trypan Blue.

### 4.8. Apoptosis

For the detection of apoptosis, the Alexa Fluor^®^ 488 Annexin V/Dead Cell Apoptosis Kit (Molecular Probes^®^, Invitrogen Carlsbad, CA, USA) was used on cells seeded in Lab-Tek™ II Chamber Slide (Thermo Scientific, Waltham, MA 02451, USA) and treated with GSE at 25, 50, 75 and 100 μg of GAE/mL of medium for 24 h. In this assay, Annexin V detects the translocation of phosphatidylserine from the inner leaflets to the outer leaflets of the plasma membrane, which is typical of apoptotic cells, whereas propidium iodide (PI) detects necrotic cells with permeabilized plasma membranes. Labelling of early apoptotic and dead cells was performed according to the manufacturer’s instructions. Fluorescent cells were immediately detected by laser scanning confocal microscope (Pascal, Carl Zeiss, Munich, Germany).

The quantitative analysis was performed by considering the florescence intensity data (mean intensity) associated to each digital image and related to the channel 1, Ch1, corresponding to the PI emission fluorescence, and to channel 2, Ch2, corresponding to AlexaFluor488 emission fluorescence. Each image was recorded by Plan-Neofluar 40×/0.75 objective, pinhole 97µm, filters Ch1: LP560, Ch2:BP505-530, laser 488 6.0% and laser 543 at 50%. A minimum of 6 images for each treatment and for each of the two independent experiments was recorded. At least 10 images per each treatment was considered. Each image captured a field corresponding to 106062,88 µm × µm of area (1048576 pixels). In order to avoid errors generated by the fluorescence methodology, a further control was performed by counting the number of stained cells in each image. The measurement of the number of cells stained with propidium iodide (PI) and Annexin V—Alexa Fluor^®^ 488—conjugated or with both the dyes (co-localization) in each of the 10–12 digital images recorded for each treatment or controls was performed. Data in both cases are the mean ± SD, *n* = 10.

### 4.9. Estimation of GJIC by Scrape-Loading/Dye-Transfer (SL/DT) Assay

Intercellular gap-junctional communication (GJIC) in MCF-7 adenocarcinoma cell cultures was assessed using the scrape-loading dye-transfer (SL/DT) technique described in [102] and [44], with modifications. Briefly, cells were seeded at 10^6^ cells/plate in 35 mm cell culture dishes, and incubated at 37 °C and 5% humidified atmosphere. Only cells at 95% confluence were used for the experiments herein; they were incubated with GSE at 25, 50, 75 and 100 μg GAE/mL of medium, for 30 min, 1 h, 2 h or 4 h. Controls containing only medium or vehicle (acetonitrile) were included in each experiment. After treatment, the cell cultures were rinsed carefully with phosphate buffer saline (PBS) without Ca^2+^ Mg^2+^, and then scraped and incubated with 0.5 mL of 0.1% (w/v) Lucifer Yellow CH (Molecular Probes, Invitrogen, Carlsbad, CA, USA), and 0.05% dextran conjugate-Rhodamine B, 10,000 MW (Invitrogen, Carlsbad, CA, USA) in Ca^2+^ Mg^2+^ free PBS, for 3 min. The cells were then washed with PBS and fixed with 4% paraformaldehyde in PBS. Gap-junction-coupled cells were imaged using a laser scanning confocal microscope (Carl Zeiss, Munich, Germany) and the percentage of cells coupled was determined in each experiment by counting fluorescent cells labelled with Lucifer Yellow, in communication via gap junctions and the cells initially loaded with LY and Rhodamine dextran at a scrape edge (co-localized dyes in injected cells). The number of communicating cells in treated samples was compared with the controls. Three random images were recorded for each plate and means were considered. Three independent experiments were performed in triplicate for each treatment and control.

### 4.10. Western Blot Analisys of Connexin 43

Cells treated for 4, 8 and 24 h with 25 μg/mL GSE and controls (untreated cells and cells treated with acetonitrile) were washed with PBS (0.01 M Na_2_HPO_4_, 0.15 M NaCl, pH 7.4), centrifuged at 5000 rpm for 5min and the pellet was collected. The pellets were suspended in 0.5 mL of 0.1 M Na-phosphate pH 6.5 and lysed by three freeze/thaw cycles. Lysate cells were suspended in Laemli buffer and proteins were extracted; 15 μg of proteins were loaded and separated on a 15% SDS-polyacrylamide gel. The Western blot was performed as described in [32] using rabbit anti-Connexin-43 antibody (Zymed) diluted 1:1000 in PBS, 3% bovine serum albumin, BSA (Sigma, Buchs, Switzerland) and ECL anti-rabbit IgG, peroxidase-linked (Invitrogen, Tharmofisher), was used as secondary antibody. The immunoreactive proteins were revealed using ECL Western blotting detection kit (BioRad, Hercules, CA, USA) following the manufacturer’s instructions. Anti-beta actin antibody was used to verify the protein amount loaded on the gel.

### 4.11. Immunohistochemistry and Confocal Microscopy

MCF-7 cells seeded on chamber slides were treated with 25 or 50 μg/mL GSE for 2 h or 24 h, and the related controls were used for immunochemistry and confocal microscopy analyses as described in [32]. Different experiments were done using both anti-Cx43 from Sigma-Aldrich and from Zymed as primary antibody (1:250 in PBS-BSA). In addition, Alexa Fluor^®^ 633-conjugated anti-rabbit IgG or Alexa Fluor^®^ 488 conjugated anti-rabbit IgG as secondary antibodies, and PI or SYBRGreen (all products from Invitrogen) as nuclear counterstain were used. Only images obtained from immunodetection with anti-Cx43 from Sigma-Aldrich, Alexa Fluor^®^ 633-conjugated anti-rabbit IgG and SYBR green are shown. Negative controls were obtained with non-immune serum followed by a secondary antibody or with primary or secondary antibodies only. A minimum of five randomly selected areas from each chamber of each GSE treatment or control were analysed by confocal microscopy (LSM Pascal, Zeiss, Munich, Germany).

### 4.12. Extraction of mRNA from MCF-7 Cells Treated with GSE

Total RNA was extracted from MCF-7 cultured cells treated with 25 or 50 μg/mL GSE for 2 h or 24 h and from the related controls, as described in [31]. cDNA was synthesized from the total RNA using a SuperScript II Reverse Transcriptase kit (Invitrogen), according to the manufacturer’s instructions.

### 4.13. Quantitative Real-Time PCR

The RT reaction was performed using 20 ng of total cDNA for each replicate in a total volume of 25 μL. The real-time reactions were performed using TaqMan^®^ Gene Expression Assay for gap junction protein alpha 1, 43kDa (assay ID: Hs00748445_s1) on a 7500 Real-Time PCR System (Applied Biosystems, Foster City, CA, USA) in 96-well plates. TaqMan Gene Expression Assay consists of two sequence-specific PCR primers and a TaqMan assay-FAM labelled MGB (minor groove binder) probe. The assay was performed in three replicates for each sample. Assays were run with 2 x Universal Master Mix without Uracil-N-glycosylase using universal cycling conditions (10 min at 95 °C; 15 s at 95 °C, 1 min 60 °C, 40 cycles). Relative quantification was performed with the SDS1.2 software (ABI), using expression of human ACTB (beta-actin) as endogenous reference control and sample treated with vehicle only as calibrator sample. The results are expressed as Log_10_ of relative quantification (RQ) calculated using the ΔΔCt method.

### 4.14. Statistical Analysis

Comparisons between treated cells and corresponding controls without GSE (only medium or vehicle) were analysed by one-way analysis of variance (ANOVA) and the Student’s t-test using Prism 7.0 (GraphPad) and statistical functions of Excel (Microsoft). Statistical significance correlations were evaluated at *p* ≤ 0.05, *p* ≤ 0.03 or *p* ≤ 0.01.

## 5. Conclusions

In conclusion, growing evidence is now substantiating the health properties of GSE and some action mechanisms are being clarified. In this work, the phenolic fraction of GSE, obtained from pomaces from the typical Negramaro grapes, highly rich in polyphenols, was able to induce apoptotic cell death in MCF-7 breast cancer cells at suitable concentrations. The pro-apoptotic effect was preceded by a transient enhancement of cell-cell communication mediated by gap junctions, which seemed due to a re-localization of Cx43 proteins on plasma membranes as well as to the induction of *cx43* mRNA expression. GJIC is a basal cellular function strictly related to the carcinogenesis process and should be considered a target for potential chemotherapeutic compounds, mainly those found in natural products. GJIC is a basal cellular function strictly related to the carcinogenesis process and apoptosis, so the control of gap-junctional permeability, even by varying Cx expression, could be considered a target for potential chemotherapeutic compounds. One of the major discrepancies between epidemiological findings of anti-cancer effects of certain dietary components of fruits and vegetables and animal, in vitro and molecular mechanistic studies might be that there are multiple signalling mechanisms that epigenetically affect GJIC. By eating whole foods, as well as food enriched with nutraceutical components, the mixtures of all the different bioactive compounds might allow for maximum signalling, whereas the isolated pure individual bioactive compound might not be able to trigger in vivo those signals needed for a physiological effect [103]. Considering that GJIC and Cxs seems related to the chemopreventive role of many dietary phytochemicals and natural compounds, the effect of complex mixtures of phytochemicals on GJIC should be considered as a screening target for isolation of bioactive natural products.

The growing attention in dietary components having nutraceutical value together with the adverse side effects of the chemotherapeutic drugs, increase the interest in plant-based active anti-cancer compounds that could provide an additional option in chemoprevention. The significance of results here presented also is related to the current growing interest in exploiting antioxidant compounds, such as polyphenols, present as dietary components or generated as by-products in the food industry.

## Figures and Tables

**Table 1:** Total phenol content and antioxidant activity of grape seed polyphenolic extracts (GSE).

GSE Samples	Total Phenols	Antioxidant Activity	
	*g of GAE/100 g GS* ± *DS*	*mol TE/GAE* ± *DS*	*mol TE/100 g GS* ± *DS*
GSE methanol:water (80:20)	1.64 ± 0.04	7.07 ± 0.54	11.6 ± 0.5
GSE ethanol:water (80:20)	0.55 ± 0.02	5.87 ± 0.70	3.3 ± 0.1

## Abbreviations

GJIC	Gap-junction-intercellular communications
Cx43	Connexin-43 protein
*cx43*	Connexin-43 gene
TEAC	Trolox Equivalent Antioxidant Capacity
SL/DT	Scrape-Loading/Dye-Transfer assay
GSE	Grape seeds extracts
GAE	Gallic Acid Equivalents
ROS	Reactive oxygen species
TE	Trolox Equivalents
GS	Grape Seeds
GJ	Gap junction
